# Striatal function scrutinized through the PAN-TAN-FSI triumvirate

**DOI:** 10.3389/fncel.2025.1572657

**Published:** 2025-03-25

**Authors:** Paul Apicella, Anne-Caroline Martel, Kevin Marche

**Affiliations:** Institut de Neurosciences de la Timone UMR 7289, Aix Marseille Université, Marseille, France

**Keywords:** basal ganglia, neuron types, behavior, single-unit, monkey

## Abstract

Understanding the information encoded by distinct components of the neuronal circuitry in the striatum represents an avenue for elucidating the role of this subcortical region in adaptive behavior and its dysfunction in pathological conditions. In behaving animals, conventional single neuron recordings generally differentiated between three main electrophysiologically identified neuron subtypes in the striatum, referred to as phasically active neurons (PANs), tonically active neurons (TANs), and fast-spiking interneurons (FSIs), assumed to correspond to GABAergic spiny projection neurons, cholinergic interneurons, and parvalbumin-containing GABAergic interneurons, respectively. Considerable research has been devoted to exploring the behavior-related activities of neurons classified electrophysiologically into PANs, TANs, and FSIs in animals engaged in task performance, mostly monkeys. Although precise neuron identification remains a major challenge, such electrophysiological studies have provided insights into the functional properties of presumed distinct striatal neuronal populations. In this review, we will focus on current ideas about the functions subserved by these neuron subtypes, emphasizing their link to specific aspects of behaviors. We will also underline the issues that are yet to be resolved regarding the classification of striatal neurons into distinct subgroups which emphasize the importance of considering the potential overlap among electrophysiological characteristics and the molecular diversity of neuron types in the striatum.

## Introduction

The striatum has been implicated in different behavioral control mechanisms, particularly those related to action selection and reward-guided learning. Efforts aimed at elucidating the role of this subcortical structure in motivated behaviors are mostly based on an examination of the coding properties of the components of the striatal circuitry in animals engaged in task performance. Considerable research has been done during the last five decades in exploring the behavior-related activity of electrophysiologically identified subtypes of neurons in the striatum of both rodents and monkeys trained to perform a variety of tasks. The single-neuron recording method has been used to study the correlation of neuronal activity with behavior, in an attempt to electrophysiologically identify neuronal populations whose properties are thought to reflect a specific function during behavior. There is broad acceptance that striatal neurons can be divided into three main categories based on electrophysiological features and much progress has been made in understanding their properties reflecting specific function during behavior. Through a better evaluation of their behavioral relationships, it is hoped that we will gain insights into the role of distinct neuronal populations in regulating the information processing within the striatal circuitry. In this review, we summarize the main ideas about the functions of the distinct types of striatal neurons recorded extracellularly in behaving animals, mostly monkeys. We conclude on the newest experimental approaches that allow for a better targeting of specific neuronal populations in combination with monitoring of their activity to disentangle the specific behavioral contribution of identified striatal neurons.

## Functional classification of neuronal types in the striatum

**Figure 1:**
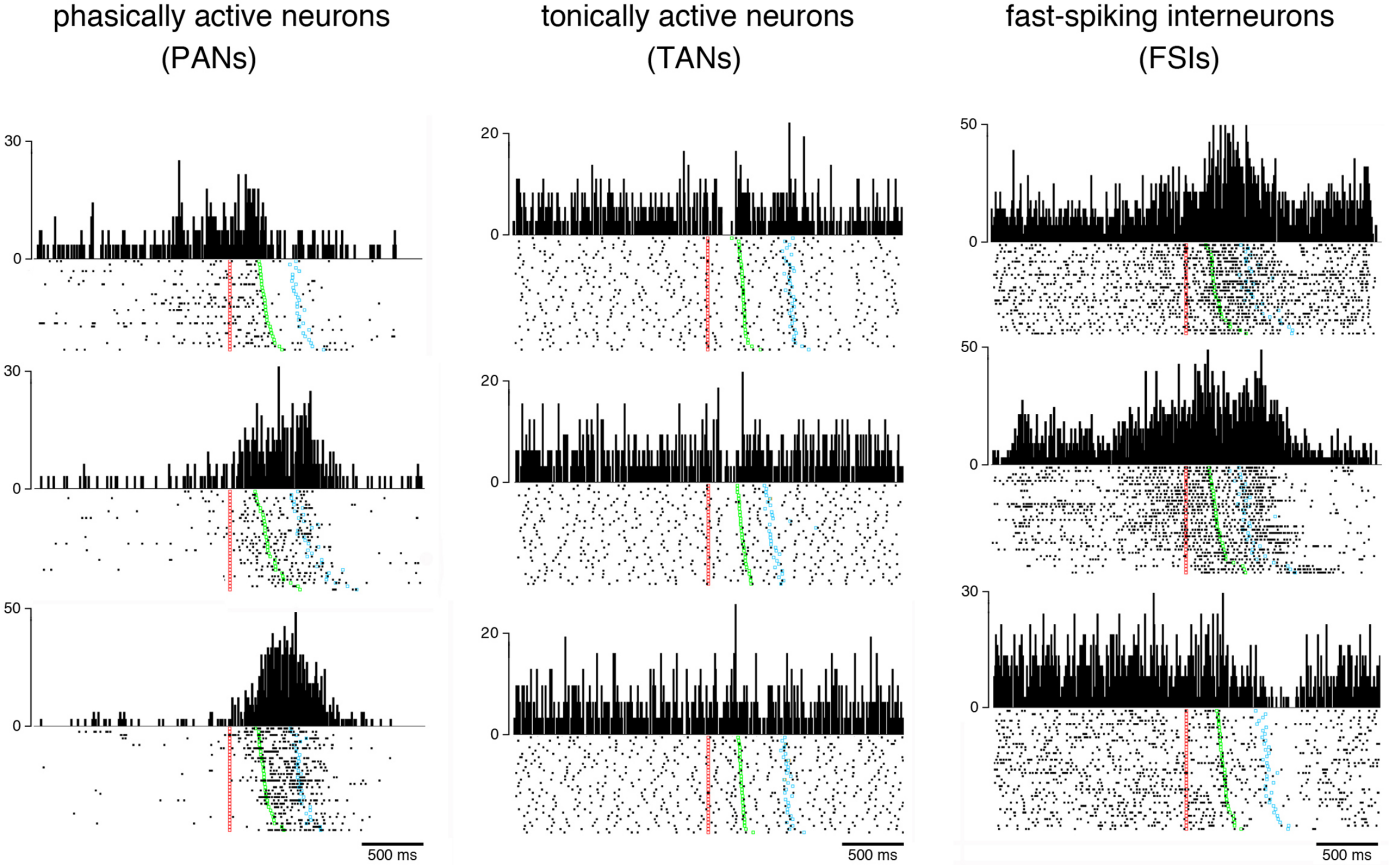
Examples of behavior-related changes in activity of the three main categories of neurons recorded in the monkey striatum. Neuronal activity is represented as raster plots (bottom) and perievent time histograms (top) during the performance of a visuomotor task in which the monkey reacted to a visual signal (red marker) by releasing a resting bar (green marker) and reaching a target (blue marker) to obtain a liquid reward. Each dot corresponds to the time of a neuronal impulse or spike and each line of dots to one trial. Raster plots are aligned on the onset of the stimulus serving as a trigger for arm-reaching movements and trials were ordered off-line according to the latency of movement. Vertical calibration is in spikes/s for all histograms. Baseline firing rates (i.e., activity during the period immediately preceding stimulus onset) vary among the three neuronal populations. PANs are usually silent or have a baseline firing rate < 1 spike/s, and display transient or sustained increases in discharge rate occurring in distinctive forms during different periods of the task, reflecting different processes that participate in movement generation. TANs fire more regularly than PANs, with firing frequencies ranging from 3 to 12 spikes/s, and exhibit quite homogeneous task-related changes in activity consisting mostly of a short lasting pause in firing in response to the visual signal. FSIs are characterized by a high firing variability with complex task-related modulations that combined increases and decreases in firing rate. Unlike TANs, FSI activity did not exhibit coordinated changes at specific moments within the context of the task used here.

In the early 1980s, electrophysiological studies in behaving monkeys began to differentiate between two main types of neurons within the striatum, termed phasically active neurons (PANs) and tonically active neurons (TANs), presumed to be GABAergic projection neurons and cholinergic interneurons, respectively (Kimura et al., 1984; Alexander and DeLong, 1985). More recent analyses of spiking features in the monkey striatum have allowed to distinguish a third class of neurons known as fast-spiking interneurons or FSIs (Adler et al., 2013; Yamada et al., 2016; Marche and Apicella, 2017; Banaie Boroujeni et al., 2020) thought to correspond to parvalbumin-containing GABAergic interneurons which have first been studied more thoroughly in rodents (Berke, 2011). Figure 1 illustrates the three electrophysiologically defined categories of neurons recorded in the striatum of the macaque monkey during task performance. Although the large majority of electrophysiological studies, particularly in monkeys, differentiated between three different neuron types within the striatum, an inability to classify neurons into distinct classes by using spontaneous firing rate combined with spike waveforms has been reported in very rare instances (Costa et al., 2019). This is, in actual fact, an unexpected result, given that PANs and TANs are the most frequently recorded and easily differentiable neurons in the monkey striatum. The reasons for the lack of separation into distinct neuron types have yet to be clarified.

Electrophysiological work with behaving rodents has also classified the neurons of the striatum into three main categories, sometimes with different labels (e.g., high-firing interneurons or HFNs, instead of FSIs), resembling the three recognized types of extracellularly recorded neuron found in the striatum of monkeys (Schmitzer-Torbert and Redish, 2008; Hernandez et al., 2013; Atallah et al., 2014; Thorn and Graybiel, 2014). The classification of striatal neurons based on electrophysiological features in behaving rodents has also allowed to distinguish a rare class of spontaneously active neuron forming a potential fourth class corresponding to low-threshold-spiking (LTS) interneurons (Berke et al., 2004; Gage et al., 2010), presumably somatostatin-expressing GABAergic interneurons. Recently, it has been suggested that putative LTS interneurons could also be identified in the monkey (Banaie Boroujeni et al., 2020). These presumed interneurons have not been extensively studied so far with electrophysiological recordings in behaving animals, due to their low number and difficulty in distinguishing them clearly (but see Holly et al., 2019).

The organization of inputs to each of the three main neuronal populations identified electrophysiologically has attracted much attention in order to gain insights into their functional properties. Each neuronal type in the striatum is under the influence of a dopaminergic input from the midbrain. It is commonly believed that the activity of PANs is dependent on their location within the striatum, based on the topography of cortico-striatal projections which determines regions of distinct functional specializations, (i.e., sensorimotor, associative and limbic). The inputs from the thalamus (intralaminar nuclei) to cholinergic TANs are assumed to be more prevalent, compared to those from the cortex. Recently, rabies tracing studies in rodents have reported that cortical inputs to FSIs (presumed parvalbumin-containing GABAergic interneurons) originate predominantly from sensorimotor areas, with little afferents from thalamus, whereas cortical inputs to TANs preferentially come from medial prefrontal areas (Klug et al., 2018). The same study showed that TANs are also under an inhibitory influence of the external globus pallidus and an excitatory influence of the pedunculopontine nucleus.

In the following subsections, we provide a brief overview of the properties of the three “classic” striatal cell populations identified electrophysiologically in behaving animals. Due to space considerations, we rely mainly on single-neuron recording studies conducted on nonhuman primates.

## Phasically active neurons

Early investigations of the activity of PANs, the most common type of neurons recorded in the striatum of awake animals, have shown that these presumed projection neurons display a large variety of activity modulations following or anticipating a task event, either sensory or motor. It has been well documented that the task-related modulations of PAN activity are linked to various processes, such as the preparation, initiation, and execution of movements. Many PANs also display activations preceding or following rewards (Apicella et al., 1991, 1992; Hikosaka et al., 1989; Schultz et al., 1992), with responses varying according to reward quality (Hassani et al., 2001; Cromwell and Schultz, 2003). Several PAN activations related to ongoing task performance may be influenced by the expectation of reward delivered at trial end, emphasizing their involvement in motivational aspects of task control (Hollerman et al., 1998; Kawagoe et al., 1998; Tremblay et al., 1998; Lauwereyns et al., 2002). In recent years, several studies have shown that PANs can be modulated by the values associated with stimuli and actions, emphasizing their role in reward-guided action selection and various forms of learning (Samejima et al., 2005; Lau and Glimcher, 2008; Ding and Gold, 2010; Cai et al., 2011; Kim and Hikosaka, 2013; Nonomura and Samejima, 2019). These studies have identified the striatum as a critical component in the brain circuitry underlying the ability to develop effective decision-making strategies based on expected value (Hikosaka et al., 2014). Some PANs were also selectively modulated by events or actions associated with outcome uncertainty (i.e., risk) (Yanike and Ferrera, 2014; White and Monosov, 2016), the encoding of uncertainty being crucial for adjusting action choices and learning. It has also been documented that the neuronal representation of time is distributed across multiple brain structures, including the striatum, with PANs encoding temporal information that guides the appropriate selection of actions in animals performing timing tasks (Chiba et al., 2015; Wang et al., 2018; Rolando et al., 2024).

This brief overview of the PAN literature in monkeys indicates that several functional aspects involved in the expression of motivated behaviors may find expression in changes in activity of PANs. However, it is not always clear whether a variation of functional properties of PANs may be related to the regional specializations within the striatum (i.e., sensorimotor, associative, and limbic territories), as a reflection of the topography of cortico-striatal connectivity. It is well established that PANs activated with body or orofacial movements are found in the dorsal part of the posterior putamen (Crutcher and DeLong, 1984; Kimura, 1990) whereas those linked to oculomotor behavior are in the head and body of the caudate nucleus (Hikosaka et al., 1989). On the other hand, reward-related changes in PAN activity are not localized exclusively in the ventral part of the anterior striatum (i.e., ventral striatum), commonly considered as a center for reward processing, but can be found distributed across the dorsal and ventral striatum (Hikosaka et al., 2006). In general, PANs showing a large variety of relationships to action valuation and action selection are intermixed in the dorsal striatum. Interestingly, PANs specialized in the detection and processing of stimuli associated with reward uncertainty have been reported to be found more often in regions of the anterior part of the dorsal striatum, close to the internal capsule (White and Monosov, 2016).

## Tonically active neurons

We and others previously found that TANs, presumed cholinergic interneurons, are primarily concerned by detecting events of motivational salience (Aosaki et al., 1994; Apicella, 2002). Although there is broad consensus that TAN signaling is critical for learning values associated with stimuli and actions, there is now evidence that the role of these neurons applies to a wider range of functions than just detecting reward-related events. In particular, studies have found that TANs can also display modulations when primary aversive stimuli are presented (Ravel et al., 2003). In addition, in spite of a lack of clear relationships to movements, other studies have documented that TANs may also be sensitive to some aspects of motor performance (Lee et al., 2006; Ravel et al., 2006; Nougaret and Ravel, 2015).

Numerous studies, using a variety of behavioral tasks in monkeys, have shown that task-related changes in TAN activity are dependent on the context of performance (Shimo and Hikosaka, 2001; Yamada et al., 2004; Lee et al., 2006; Ravel et al., 2006; Martel and Apicella, 2024). Therefore, increasing attention has been paid to the role of TANs in the integration of contextual information within the striatal circuitry (Apicella, 2007). Growing evidence suggests that TANs may provide signals potentially suitable for the switching of behavior based on changing conditions and contribute to the representation of contextual features of the environment in which learning and actions occur (Bradfield et al., 2013), emphasizing their implication in mediating the flexibility of behavior. Recordings from rodents have provided support to the hypothesis that TANs emit signals potentially suitable for the recognition of a context for learning and action selection (Stalnaker et al., 2016). However, further investigations are needed in behaving animals, including monkeys, to validate the hypothesis that the TAN system may keep track of context.

Although behavior-related modulations of TAN activity are generally described as homogeneous throughout the striatum, region-specific properties of TAN activity have been reported in behaving rats (Thorn and Graybiel, 2014; Stalnaker et al., 2016) and monkeys (Yamada et al., 2004; Marche et al., 2017). A variation of functional properties of TANs depending on the regional specializations of the striatum is still an open question.

## Fast-spiking interneurons

Much of our knowledge about the functional properties of FSIs has been derived from rodent work. Several studies have provided evidence in support of a role of these presumed GABAergic interneurons in action selection and movement execution (Schmitzer-Torbert and Redish, 2008; Gage et al., 2010; Stalnaker et al., 2012; Kim et al., 2014; Bakhurin et al., 2016; Kulik et al., 2017). Other data have suggested that FSIs in the ventral striatum (i.e., nucleus accumbens) may provide signals related to expectation and receipt of rewards (Lansink et al., 2010; Atallah et al., 2014), indicating that changes in FSIs activity may be variable depending on striatal regions. It was also reported that FSI activity modulation changed over reward-guided learning, with the dynamics of these changes being specific of the striatal region (Thorn and Graybiel, 2014).

Until now, limited data are available regarding the behavioral contributions of FSIs in monkeys because they appear to be less frequently recorded compared to PANs and TANs. Early studies in behaving monkeys have suggested a possible role of FSIs in the encoding of action and outcome (Adler et al., 2013; Yamada et al., 2016). When studying FSI activity during performance of a visuomotor task, we have found that these neurons may display changes in activity before and during the movement, with complex time courses combining increases and decreases in firing rate (Marche and Apicella, 2017). Our work further suggested that the modulation of FSI activity around movement onset could be dependent on the mode of movement selection (i.e., internally or externally-instructed movements), suggesting that these neurons are influenced by the context of motor performance (Marche and Apicella, 2021). Recent work has pointed to changes in FSI activity in the most posterior part of the striatum (i.e., striatum tail) related to contextual factors (Kunimatsu et al., 2021), suggesting that these neurons contribute to adjusting choice behavior when the context is modified. Another recent study in monkeys has demonstrated that FSIs recorded in the anterior striatum, including the caudate nucleus and ventral striatum, play a role in attention and learning processes (Banaie Boroujeni et al., 2020). This latter study revealed two subtypes of FSIs whose activity was differentially modulated during and after learning the value of stimuli, with some FSIs being preferentially activated during the acquisition phase of training, while other FSIs became inactive later when the reward association of the attention cue is learned (Banaie Boroujeni et al., 2020). At the moment, it is still difficult to get a clear picture of the relationship between FSI activity and behavioral variables, the precise nature of the information conveyed by FSIs and its relevance to striatal functions being a matter of debate. Additional research is clearly required to further characterize the properties of FSI signals to gain more detailed insights into the way in which these neurons work within the striatal circuitry.

We will now discuss the limitations in current approaches to the identification that can be made from extracellular recordings, particularly in primates, and the difficulty to specifically target striatal neuron types for physiological investigations in animals engaged in task performance.

## Challenging issues in the classification of striatal neurons into distinct categories

Electrophysiological criteria for distinguishing PANs, TANs, and FSIs have become accepted as indirect markers of neuronal identity and are currently used in most rodent and monkey studies to investigate the contributions of specific cell populations in the striatum to different aspects of behavior. Nevertheless, the relevance of this categorization is debated with regards to the variety of striatal neurons which have been characterized, particularly at the level of local GABAergic microcircuits (Tepper et al., 2010; Silberberg and Bolam, 2015), and the difficulty in accurately distinguishing striatal cell types using electrophysiological criteria. Although PANs, TANs, and FSIs are usually considered as single functionally-homogenous populations, each of these categories may actually exhibit greater diversity than currently recognized. It therefore becomes important to take into account heterogeneity within the different populations of striatal neurons identified electrophysiologically in awake animals. Challenges in targeting specific neuronal subtypes can be overcome by using complementary methods for the identification of extracellularly recorded neurons, such as optogenetic tagging which has proven to be effective in confirming neuron identity in the striatum of genetically engineered rodents engaged in task performance (Atallah et al., 2014; Duhne et al., 2024). However, experimental approaches combining electrophysiological recordings and genetic tools to label and target specific neurons for extracellular recording are still difficult to implement in primates.

Recently, studies in both rodents and primates have employed powerful methods (i.e., transcriptomics analysis) for the identification of neuron subtypes according to their gene expression patterns. Based on these findings, it is now possible to classify and characterize striatal neurons into molecularly distinct subgroups in both rodents and primates (Gokce et al., 2016; Munoz-Manchado et al., 2018; Martin et al., 2019; Krienen et al., 2020; Stanley et al., 2020; He et al., 2021). Studies using molecular identification methods combined with photometry recordings of neuronal activity (i.e., calcium imaging) have been used in behaving rodents to examine neuronal signals from the so-called striosome and matrix compartments of the dorsal striatum that have long been remained indistinguishable for recording studies during behavioral performance (Friedman et al., 2020). Systematic studies of genetically tagged striatal neurons in behaving animals combined with electrophysiology or calcium imaging of identified neuron subtypes represent a major advance in investigating the role of the various components of the striatal circuitry.

## Conclusion

We have reviewed briefly single-neuron recording studies examining the neuronal bases of behavior at the level of three main classes of neurons that have traditionally be recognized in the striatum of behaving animals. Although the exact identity of extracellularly recorded neurons remains questionable, this approach has provided insights into the functional properties of presumed distinct neuronal populations in the striatum. Recently, the use of neuron-type-specific analyses has revolutionized physiological investigations of striatal function making their continued use essential for future experiments. However, there are technical difficulties in reliably identifying neuron subtypes during behavior, and one can expect further advances in this direction in coming years. Notably, experiments with non-human primates currently lag behind rodents studies in their ability to precisely target specific neuron types in the striatum.

A more detailed classification of striatal neurons in rodents and monkeys is relevant to address the issue of possible differences between species. In some instances, the data gathered in rodents are not fully in agreement with those collected in monkeys, raising questions about neuron-type homologies between rodents and primates. In primates, the proportion of striatal GABAergic interneurons has been reported to be greater than in rodents (Wu and Parent, 2000). Phylogenetic variation in the organization of the striatal circuitry could have implications for information processing and more caution may be required when translating findings between rodents and primates. In addition, greater consideration of the heterogeneity of striatal neurons can help refine theoretical models of striatal function by incorporating diverse components of the striatal circuitry. This approach will enhance our understanding of the neuronal mechanisms underlying reward-based learning and action selection in the striatum.

Finally, methods for investigating and analyzing the function of the different components of striatal circuitry are important for unraveling the mechanisms mediating normal behavior and its disruption in pathological conditions. Future studies focusing on circuits specified by functional cell type composition may have implications for understanding behavioral disturbances in patients with neurological and psychiatric disorders, such as Parkinson’s disease or compulsive behaviors. These findings could lead to the potential development of targeted pharmacological treatments of these striatal-based disorders.
